# In Vitro Characterization of Cisplatin and Pemetrexed Effects in Malignant Pleural Mesothelioma 3D Culture Phenotypes

**DOI:** 10.3390/cancers11101446

**Published:** 2019-09-27

**Authors:** Eleftherios D. Papazoglou, Rajesh M. Jagirdar, Olympia A. Kouliou, Eleanna Pitaraki, Chrissi Hatzoglou, Konstantinos I. Gourgoulianis, Sotirios G. Zarogiannis

**Affiliations:** 1 Department of Physiology, Faculty of Medicine, University of Thessaly, Biopolis, 41500 Larissa, Greece; epapazoglou@uth.gr (E.D.P.); raj.jagirdar@gmail.com (R.M.J.); okouliou@uth.gr (O.A.K.); epitaraki@uth.gr (E.P.); chatz@med.uth.gr (C.H.); 2 Department of Respiratory Medicine, Faculty of Medicine, University of Thessaly, Biopolis, 41500 Larissa, Greece; kgourg@med.uth.gr

**Keywords:** 3D cultures, cisplatin, homologous cell derived extracellular matrix, malignant pleural mesothelioma, mesothelial cells, pemetrexed, pleura, tumor spheroid

## Abstract

Malignant pleural mesothelioma (MPM) is an aggressive cancer with poor prognosis. The main treatment for MPM is doublet chemotherapy with Cisplatin and Pemetrexed, while ongoing trials test the efficacy of pemetrexed monotherapy. However, there is lack of evidence regarding the effects of Cisplatin and Pemetrexed on MPM cell phenotypes, especially in three-dimensional (3D) cell cultures. In this study, we evaluated the effects Cisplatin and Pemetrexed on cell viability using homologous cell derived extracellular matrix (hECM) as substratum and subsequently in the following 3D cell culture phenotypes: tumor spheroid formation, tumor spheroid invasion, and collagen gel contraction. We used benign mesothelial MeT-5A cells as controls and the MPM cell lines M14K (epithelioid), MSTO (biphasic), and ZL34 (sarcomatoid). Cell viability of all cell lines was significantly decreased with all treatments. Mean tumor spheroid perimeter was reduced after treatment with Pemetrexed or the doublet therapy in all cell lines, while Cisplatin reduced the mean spheroid perimeter of MeT-5A and MSTO cells. Doublet treatment reduced the invasive capacity of spheroids of cell lines into collagenous matrices, while Cisplatin lowered the invasion of the MSTO and ZL34 cell lines, and Pemetrexed lowered the invasion of MeT-5A and ZL34 cell lines. Treatment with Pemetrexed or the combination significantly reduced the collagen gel contraction of all cell lines, while Cisplatin treatment affected only the MeT-5A and M14K cells. The results of the current study can be used as an in vitro 3D platform for testing novel drugs against MPM for ameliorating the effects of first line chemotherapeutics.

## 1. Introduction

Malignant Pleural Mesothelioma (MPM) is a rare and fatal cancer causatively linked to asbestos exposure that arises from the pleural mesothelium. MPM cases comprise 80% of the total mesothelioma cases that may also occur in the peritoneum and the pericardium [1]. Despite asbestos being a regulated material in many countries, there are several other similar, fibrous materials that have been linked to MPM development [2]. These materials could potentially prolong the asbestos-induced MPM epidemic, which is expected to reach its peak in around 2020 [2]. The median survival of MPM patients is 12 months following diagnosis and the treatment options (chemotherapy, radiotherapy, surgery and palliative therapy) depend on the stage of the disease. Since MPM is asymptomatic at early stages, most patients are diagnosed at stage III, and thus cytoreductive surgery is usually not applicable [3]. Chemotherapy comprises the main therapy option for MPM patients or is used as a follow-up therapy after surgery [4,5]. The front-line chemotherapy is a combination of Cisplatin (cis-diamminedichloroplatinum; CPDD) and Pemetrexed (disodium salt of a synthetic pyrimidine-based antifolate; Pem) at doses of 75 mg/m² and 500 mg/m², respectively, since it has been shown to confer a longer survival advantage [4,6]. CPDD crosslinks with the DNA purine bases, interferes with DNA repair mechanisms, and causes DNA damage, thus leading to cell apoptosis [7]. Pem inhibits three enzymes critical for the purine and pyrimidine synthesis and the folate metabolism [8]. MPM severity influences treatment response and depends on the histological subtype; sarcomatoid, biphasic, and epithelioid MPM, with sarcomatoid MPM being the most aggressive and epithelioid MPM being the least aggressive [9].

Pleural mesothelial cells undergo Epithelial-Mesenchymal Transition (EMT) during MPM development [10]. EMT is a process that renders epithelial cells with mesenchymal properties during cancer development and leads to more aggressive phenotypes. The components of the extracellular matrix (ECM) that are produced by cells are critical for EMT and tumor metastasis [11]. Moreover, a modified ECM composition due to malignant cells can induce favorable characteristics in cancer cells such as chemoresistance [12]. In physiological conditions, ECM is critical for cell homeostasis providing the proper three-dimensional (3D) tissue architecture wherein the cells can thrive; therefore the ECM component should be taken into account in the in vitro study of cell phenotypes pertinent to tumor development and progression [1]. Furthermore, cells cultured in 3D exhibit a different gene expression pattern of genes involved in cell signaling, proliferation, adhesion and apoptosis pathways as compared to monolayer (2D) cell cultures [13]. In line with the above, investigations of cancer cell phenotypes in the context of administered chemotherapeutics, would yield the most clinically relevant results when assessed in 3D cultures with the use of hECM [14].

In terms of MPM and CPDD/Pem doublet treatment, there is a scarcity of data that demonstrate the effects of the treatment on 3D cultures. In order to fill this gap in the literature, we investigated the effects of CPDD, Pem and CPDD/Pem doublet treatment on the ability of MPM cells to form tumor spheroids, on the invasiveness of these tumor spheroids and on the property of the MPM cells to contract in a 3D collagen gel environment.

## 2. Results

### 2.1. Cell Viability Was Significantly Decreased after All Treatments in all Cell Types on Homologous Cell Derived ECM Substratum

**Figure 1:**
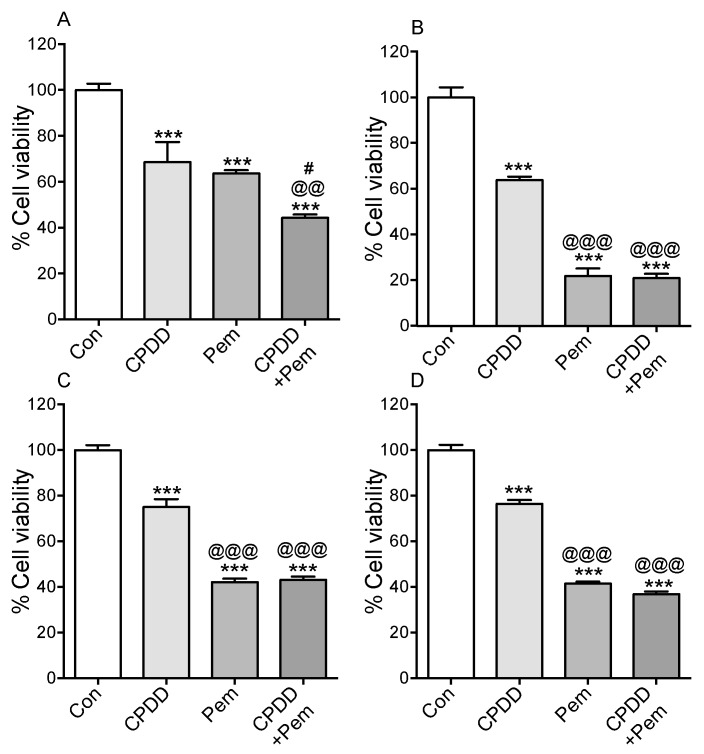
Mean values of cell viability ± SEM without or with treatments (CPDD, Pem, or CPDD+Pem) expressed as % of Controls (cells with 10% FBS-RPMI). (**A**) MeT-5A cells (*n* = 13), (**B**) M14K cells (*n* = 16), (**C**) MSTO cells (*n* = 16), (**D**) ZL34 (*n* = 15 or 16). *** *p* < 0.001 vs. Controls, ^@@^ *p* < 0.01 and ^@@@^ *p* < 0.001 vs. CPDD, ^#^ *p* < 0.05 vs. Pem.

Cells were subject to CPDD, Pem or CPDD+Pem treatments in order to assess their survival (Figure 1). 10% FBS-RPMI was used as control (Con). Cell viability with treatments was compared to control that was arbitrarily set as 100%. In MeT-5A cells, the mean cell viability was significantly lower with all drug treatments, and CPDD+Pem significantly reduced the cell viability compared to Pem or CPDD. (Con: 100.0 ± 2.75%, CPDD: 68.54 ± 8.80%, Pem: 63.64 ± 1.39%, CPDD+Pem: 44.21 ± 1.42%; *p* < 0.001). In M14K cells, the cell viability was significantly reduced by all drug treatments, while Pem treated groups had significantly reduced cell viability compared to CPDD alone (Con: 100.0 ± 4.42%, CPDD: 63.83 ± 1.45%, Pem: 21.84 ± 3.34%, CPDD+Pem: 21.02 ± 1.79%; *p* < 0.001). In MSTO cells, the mean cell viability was significantly reduced by all drug treatments. Again, Pem treated groups had significantly reduced cell viability compared to CPDD (Con: 100.0 ± 2.14%, CPDD: 74.99 ± 3.48%, Pem: 42.13 ± 1.50%, CPDD+Pem: 43.14 ± 1.39%; *p* < 0.001). Finally, in ZL34 cells the viability was significantly lowered with all drug treatments, and also in this case, Pem treated groups had significantly reduced cell viability compared to CPDD (Con: 100.0 ± 2.26%, CPDD: 76.42 ± 1.69%, Pem: 41.43 ± 0.92%, CPDD+Pem: 36.86 ± 1.15%; *p* < 0.001).

### 2.2. Tumor Spheroid Formation in All Cell Types Was Significantly Reduced with CPDD and Pem Combined Treatment

**Figure 2:**
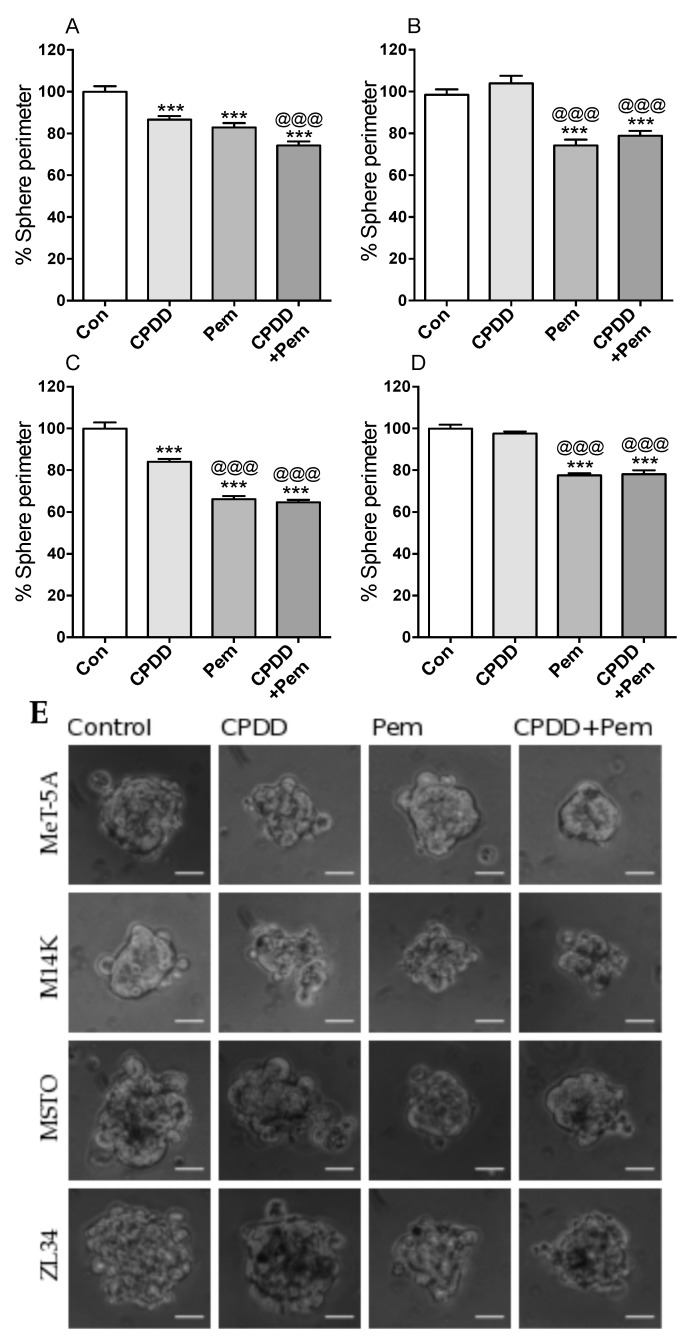
Mean values of spheroid perimeter ± SEM without or with treatments (CPDD, Pem, or CPDD+Pem) expressed as % of Controls (cells with 10% FBS-RPMI), along with representative microscopy images from each group. (**A**) MeT-5A cells (*n* = 13–17), (**B**) M14K cells (*n* = 10–15), (**C**) MSTO cells (*n* = 15–18), (**D**) ZL34 (*n* = 13–15). *** *p* < 0.001 vs. Controls, ^@@@^ *p* < 0.001 vs. CPDD. (**E**) Images of spheroids of cell types arranged by rows and their corresponding treatments in a column wise manner. Scale bars represent a length of 100 pixels.

Spheroids were formed using the hanging drop method in culture media containing drugs and comparisons were made regarding spheroid perimeter (in pixels). The mean perimeter of the spheroids formed using culture media without drugs was arbitrarily set as 100% for each cell line (Figure 2). In MeT-5A cells, the mean spheroid perimeter was significantly reduced with all drug treatments, while CPDD+Pem treatment significantly reduced the mean spheroid perimeter compared to CPDD alone but not Pem alone (Con: 100.0 ± 2.62%, CPDD: 86.63 ± 1.73%, Pem: 82.86 ± 2.15%, CPDD+Pem: 74.23 ± 2.01%; *p* < 0.001). In M14K cells, the mean spheroid perimeter was significantly reduced in the Pem treated groups (Con: 98.44 ± 2.56%, CPDD: 104.0 ± 3.57%, Pem: 74.25 ± 2.70%, CPDD+Pem: 78.87 ± 2.33%; *p* < 0.001). In MSTO cells, the mean spheroid perimeter was significantly reduced with all drug treatments, while Pem-treated groups had reduced mean spheroid perimeter significantly more compared to CPDD (Con: 100 ± 2.88%, CPDD: 84.05 ± 1.39%, Pem: 66.22 ± 1.49%, CPDD+Pem: 64.69 ± 1.13%; *p* < 0.001). Finally, in ZL34 cells as in M14K cells, the mean spheroid perimeter was significantly reduced in the Pem treated groups (Con: 100 ± 1.81%, CPDD: 97.55 ± 1.04%, Pem: 77.58 ± 0.99%, CPDD+Pem: 78.10 ± 1.87%; *p* < 0.001).

### 2.3. CPDD and Pem Combination Significantly Reduced the Invasion of MPM Spheroids in Collagen Gel Matrix

**Figure 3:**
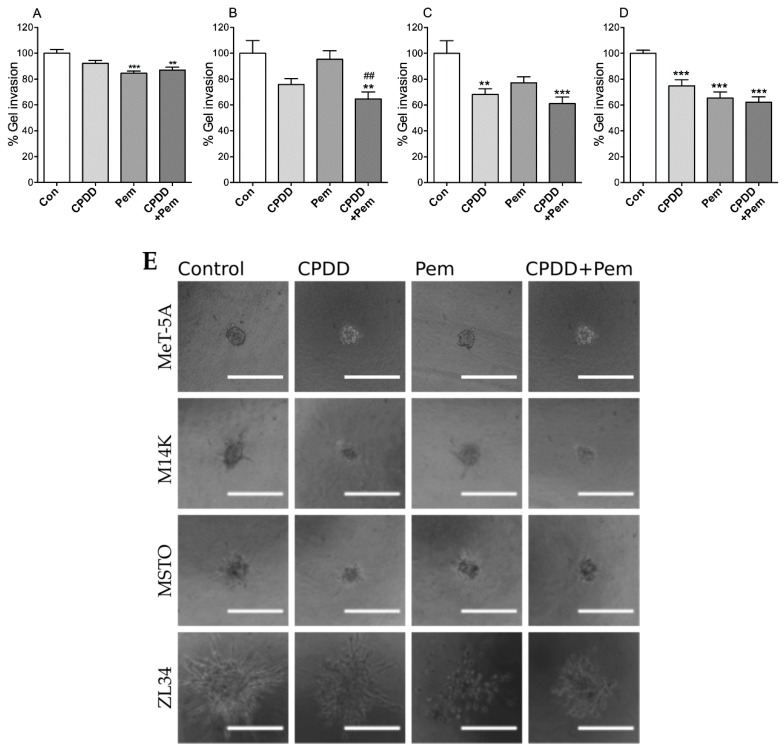
Mean values of perimeter of spheroid invasion ± SEM without or with treatments (CPDD, Pem, or CPDD+Pem) expressed as % of Controls (cells with 10% FBS-RPMI) along with representative microscopy images from each group. (**A**) MeT-5A cells (*n* = 16–21), (**B**) M14K cells (*n* = 19–22), (**C**) MSTO cells (*n* = 13–19), (**D**) ZL34 (*n* = 15–23). ** *p* < 0.01 and *** *p* < 0.001 vs. Controls, ^##^ *p* < 0.01 vs. Pem. (**E**) Images of spheroids invading into the surrounding collagen matrix, arranged by rows (by cell type) and their corresponding treatments in a column wise manner. Scale bars represent a length of 100 pixels.

MPM spheroids were embedded in collagen to evaluate the effects of CPDD and Pem during invasion inside collagen gel matrices. The mean perimeter of the spheroids inside the collagen with 10% FBS-RPMI was arbitrarily set as 100% for each untreated cell line individually (Figure 3). The MeT-5A cell spheroid invasion inside the collagen matrix was significantly lower in the Pem-treated groups (Con: 100.0 ± 3.01%, CPDD: 92.27 ± 2.20%, Pem: 84.51 ± 1.72%, CPDD+Pem: 86.98 ± 2.19%; *p* < 0.001). The M14K invasion inside the collagen matrix was significantly reduced when treated with CPDD+Pem, and CPDD+Pem significantly reduced the invasion compared to Pem (Con: 100.0 ± 9.76%, CPDD: 75.93 ± 4.40%, Pem: 95.3 ± 6.63%, CPDD+Pem: 64.61 ± 5.41%; *p* < 0.001). The MSTO cells invaded significantly less when treated with CPDD or CPDD+Pem (Con: 100.0 ± 9.79%, CPDD: 68.20 ± 4.47%, Pem: 77.28 ± 4.60%, CPDD+Pem: 61.16 ± 5.00%; *p* < 0.001). Finally, the ZL34 cells invaded significantly less with all drug treatments (Con: 100.0 ± 2.36%, CPDD: 74.87 ± 4.78%, Pem: 65.32 ± 4.77%, CPDD+Pem: 62.25 ± 4.10%; *p* < 0.001).

### 2.4. Pem Alone and CPDD and Pem Combination Significantly Reduced the Contraction of Collagen Gels

**Figure 4:**
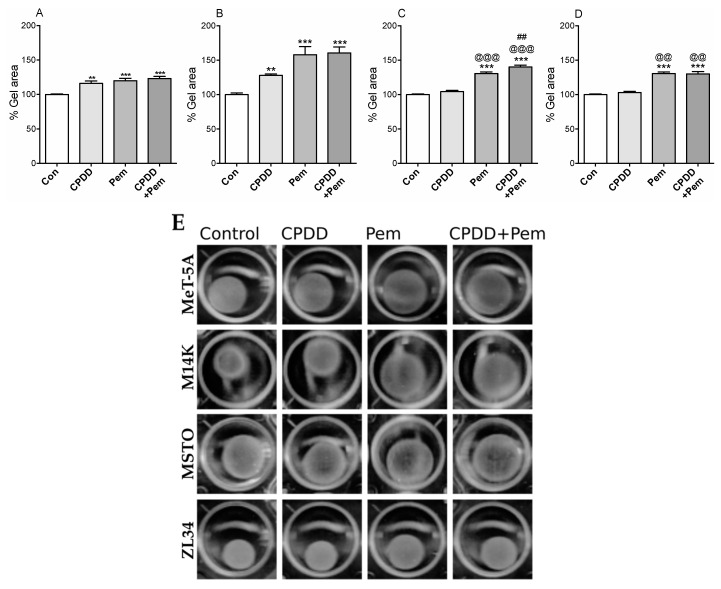
Mean values of gel area ± SEM without or with treatments (CPDD, Pem, or CPDD+Pem) expressed as % of Controls (cells with 10% FBS-RPMI) along with representative microscopy images from each group. (**A**) MeT-5A cells (*n* = 12), (**B**) M14K cells (*n* = 12), (**C**) MSTO cells (*n* = 16), (**D**) ZL34 (*n* = 11–12). ** *p* < 0.01 and *** *p* < 0.001 vs. Controls, ^@@^ *p* < 0.01 and ^@@@^ *p* < 0.001 vs. CPPD, ^##^ *p* < 0.01 vs. Pem. (**E**) Images of contracted gels with cell types arranged by rows and their corresponding treatments in a column wise manner.

Epithelial-mesenchymal transition (EMT) in 3D is a phenotype that can be studied with cells embedded in collagen gels. Cells progressively organize and contract the collagen gel post polymerization within 30 min. We performed 3D EMT studies with/without the drugs, and the final gel areas were evaluated as a measure of 3D EMT. Gel contraction with drugs was compared to cells treated with 10% FBS-RPMI (100%) (Figure 4). Regarding MeT-5A and M14K, the mean gel area was significantly higher with all treatments (MeT-5A: Con: 100.0 ± 0.94%, CPDD: 116.1 ± 3.54%, Pem: 119.8 ± 3.64%, CPDD+Pem: 123.0 ± 3.28%; *p* < 0.001), (M14K: Con: 100.0 ± 2.31%, CPDD: 127.9 ± 2.08%, Pem: 157.9 ± 11.73%, CPDD+Pem: 160.3 ± 8.63%; *p* < 0.001). In MSTO cell line, the mean gel area was significantly increased when treated with Pem or CPDD+Pem. Compared to CPDD, both Pem and CPDD+Pem significantly reduced the contraction of MSTO cells, while CPDD+Pem was more potent than Pem alone (Con: 100.0 ± 1.03%, CPDD: 104.4 ± 1.76%, Pem: 130.8 ± 2.29%, CPDD+Pem: 140.2 ± 2.66%; *p* < 0.001). In ZL34 cell line, the mean gel area was significantly higher in the Pem-treated groups compared to control or CPDD alone (Con: 100.0 ± 1.14%, CPDD: 103.2 ± 1.56%, Pem: 130.8 ± 1.93%, CPDD+Pem: 130.1 ± 3.39%; *p* < 0.001).

## 3. Discussion

In this paper, we opted to create a well characterized in vitro 3D platform for assessing the efficacy of the current chemotherapeutics used for the treatment of MPM that can be further used in order to assess adjuvant treatments. Under the scope of the above, we evaluated the effects of CPDD and Pem in the context of MPM cell viability, tumor spheroid formation, tumor spheroid invasion, and collagen gel contraction in MeT-5A, M14K, MSTO, and ZL34 cell lines. Treatment with CPDD, Pem or CPDD+Pem reduced the cell viability of all cell types as expected, but Pem or CPDD+Pem was more potent at reducing MPM cell viability as compared to CPDD treatment alone. The cytotoxic effects of CPDD and Pem on MSTO cells have been previously shown, demonstrating lower cell viability [15]. CPDD has been reported to lower the viability of MeT-5A and MSTO cells at a dose of 20 μM over 24 h on poly-L-lysine treated plates [16]. In our experiments, we used 10 μM of CPDD over 48 h on cells deposited on homologous cell-derived ECM. ECM derived from invasive cells induces 5-flouorouracil (5-FU) resistance in HT-29 colorectal cancer cells when compared to HT-29 cells that were deposited on cell-derived ECM either from non-invasive or from normal colorectal cells [17]. Moreover, based on the origin of cell-derived ECM, cells exhibit different gene expression levels of ABCB1 and ABCC1 that are involved in efflux of 5-FU [17]. Thus, the use of homologous cell-derived ECM for cell viability assays of malignant cells is a better representation of the in vivo tumor ECM architecture as it entails potential involvement of drug resistance. Our results show that, in 2D flat surfaces, Pem is the main driver of cytotoxicity in malignant cell lines, while in benign mesothelial cells, there is a synergistic effect of CPDD and Pem. During 3D growth, 3D invasion, and 3D EMT, our data shows that CPDD mono-treatment demonstrated context dependent inhibition, while in most cases CPDD+Pem was not superior to Pem alone.

With respect to spheroid formation, all treatments reduced the perimeter of MeT-5A and MSTO spheroids, while M14K and ZL34 spheroids were smaller only when treated with Pem or the combination of the drugs. The importance of tumor spheroids in MPM studies has been highlighted before. Spheroids have been shown to differentially express genes as compared to monolayers, so the use of 3D cultures demonstrates more accurately the response to drug treatments, mimicking the conditions of the human body to a better extent [13]. Through the application of the in vitro spheroid formation assay, the phenotypes of cell adhesion and cell proliferation are concomitantly assessed in an arrangement that resembles the spheroid found in pleural fluids of patients with MPM. We have shown that spheroids from all cell lines were affected by Pem treatments (Pem or CPDD+Pem), while CPDD did not reduce the perimeter of the spheroids of M14K and ZL34 cells. This suggests that the process of spheroid formation of M14K and ZL34 cells is resistant to CPDD treatment. This finding is in agreement with a previous study showing that populations of H28, H2052 (sarcomatoid MPM), and ACC-Meso4 (epithelioid MPM) cells that were expressing high concentrations of aldehyde dehydrogenase (ALDH) and were CD44 positive developed spheroids despite of CPDD treatment [18]. Based on the above, CPDD seems to affect spheroid formation in a histotype-dependent manner, while Pem was able to pose its effects regardless of the cell type, rendering Pem the critical factor for reduction of the spheroid formation in MPM patients. Furthermore, we have recently published that adding 2-deoxy-glucose (2-DG) to doublet treatment CPDD+Pem had additive effects in terms of proliferation inhibition only in 2D cultures and not in 3D spheroids in MSTO and ZL34 cells lines, which is suggestive of a significant challenge posed in the treatment of 3D tissue structures [19]. On the other hand, a recent study using the FAK inhibitor BI853520 (1 μM) showed that MPM 3D spheroid growth was lowered by FAK inhibition while this was not the case in 2D cultures [20]. Collectively, these data point to the importance of the adoption of 3D cultures in MPM treatment testing, and recently, the use of patient derived 3D organoid cultures for individual drug testing has been published [21].

Tumor spheroid invasion is a 3D assay that demonstrates the invasion potential of tumor spheroids within in a collagenous matrix. CPDD reduced the invasion of MSTO and ZL34 cells, Pem affected MeT-5A and ZL34 cells, while the combined treatment significantly reduced the spheroid invasion of spheroids from all cell lines. Collagen drop-drug sensitivity test (CD-DST) is a method evaluating the effectiveness of a drug treatment on biopsies of malignant tissues focusing on their invasiveness. CD-DST with CPDD has been reported, but the effects of combination of CPDD and Pem on different MPM histotypes are unknown [22]. Therefore, the methodology employed in the current paper demonstrates that, in the scope of the limited availability of patient biopsies, a collagen tumor spheroid invasion assay using chemotherapeutics would be a valuable tool to assess patient-specific drug effectiveness in the clinical management of mesothelioma. To further substantiate this, three recent studies have also recently shown that pirfenidone and erlotinib+photodynamic therapy were able to inhibit the 3D growth of MPM spheroids in collagen I gel and Matrigel, respectively [23,24]. Matrigel is a mouse sarcoma derived ECM that may induce other pathways implicated in cell–ECM signaling due to its cancerous origin, but still is an important tool in such studies. However, using either commercially available collagen or in-house collagen, as was done in our study, provides a better representation of the benign nature of the surrounding ECM.

Gel contraction is a 3D assay that assesses the mesenchymal potential of cancer cells by testing the contraction of malignant cells inside an ECM component, as is the case in tumor progression. The gel contraction assay is a measure of the degree of native mesenchymal transition of the cells (as measured from the area of the contracted gels). In our model, we used rat tail collagen gel areas resulting from contraction over a period of 24 h. Pem decreased the contraction of cells embedded in collagen gels in all cell lines tested. CPDD only decreased the contraction of MeT-5A and M14K cells, while the combined treatment of CPDD and Pem decreased the gel contraction in all cell lines. It has been reported that mouse uterine mesothelial cells that have undergone EMT express α-smooth muscle actin (α-SMA) and develop a contractile phenotype typical of smooth muscle cells [25]. Our cells demonstrated contractile characteristics that were inhibited with the use of chemotherapeutics.

## 4. Materials and Methods

### 4.1. Cell Culture

The human cell lines MeT-5A (benign immortalized mesothelial cells), M14K (epithelioid MPM), MSTO (biphasic MPM) and ZL34 (sarcomatoid MPM) were used and were kindly provided by Prof. Ioannis Kalomenidis (National and Kapodistrian University of Athens, Athens, Greece) [14]. Cells were cultured with 10% Fetal Bovine Serum-RPMI (F0804, Sigma, St Louis, MO, USA), 2 mM L-Glutamine (G7513, Sigma), 1% Penicillin/Streptomycin (P4333, Sigma), and 0.5% w/v Plasmocin (ANT-MPP, InvivoGen, Toulouse, France) in a 5% CO_2_ incubator. Cells were synchronized by serum starvation (0.5% FBS-RPMI) over 24 h prior to all experiments.

### 4.2. Drug Treatments

Filter sterilized 10^−5^ M Cisplatin (CPDD; PHR1624, Sigma) and 2 × 10^−4^ M Pemetrexed (Pem; CDS024404, Sigma) and their combination (CPDD+Pem) were used.

### 4.3. Acid Soluble Rat-Tail Collagen Preparation

Tails were obtained from euthanized white Norwegian Sprague-Dawley rats that were designated for disposal (kindly provided by Dr. Konstantinos Dimas, Faculty of Medicine, University of Thessaly, Larissa, Greece). The tails were cut along the anterior-posterior axis at regular intervals without penetrating the core of the tail. Small 1 cm portions of intact tail were pulled apart until cords of white material in-between were stretched. Such strings of collagen were collected, weighed and immersed in 95% ethanol and then air dried in a laminar flow hood. The dried collagen cords were subject to leaching with sterile 0.2 M acetic acid, in a sterile conical flask on a magnetic stir plate for 16 h at 4 °C. The solution was then subject to clarification by centrifuging at 2500 g for 20 min, two times in order to sediment un-dissolved protein. The resulting collagenous material was then stored in sterilized glass bottles at 4 °C.

### 4.4. Cell Viability Assay

Cells (5 × 10^4^ cells/mL) were seeded in 0.5% FBS-RPMI media for 24 h in 96-well plates pre-treated with homologous cell-derived ECM as described previously [14]. Afterward, cells were incubated for 48 h with 10% FBS-RPMI with the appropriate drugs. At the end of the assay, cells were washed with PBS, followed by fixation with 4% paraformaldehyde (PFA), then stained with 0.5% crystal violet and subsequently washed in running tap water. Plates were de-stained with 10% acetic acid after drying overnight. The acetic acid/dye solution was then subjected to O.D. measurements at 570 nm. Each experiment had a replicate of 6–8 and was repeated two times.

### 4.5. Tumor Spheroid Formation Assay

Fifty synchronized cells in 25 μL of media were used to form spheroids in suspension in a hanging drop format over 48 h. In some suspensions CPDD, Pem, or CPDD+Pem were included in the cell medium. Spheroids were imaged, and their perimeters were measured with ImageJ software using the polygon lasso tool. Each experiment had a replicate of 5–9 and was repeated two times.

### 4.6. Tumor Spheroid Invasion Assay

Spheroids were formed as described above, and then pH neutral collagen mix with 10% FBS-RPMI was overlaid on top of the spheroids. Some of the collagen-RPMI mixtures contained CPDD, Pem, or CPDD+Pem and cultured for 48 h. Spheroids inside the collagen were imaged, and their perimeters were measured using ImageJ software with the polygon lasso tool. Each experiment had a replicate of 7–12 and was repeated two times.

### 4.7. Gel Contraction Assay

All reagents were kept on ice. Neutralized collagen (1.2 to 1.5 mg/mL) with 0.5 M NaOH and 1 × 10^6^ synchronized cells/mL was used. The cells were embedded in 0.6 mL volume in 24-well plates. In some of the mixture, drugs were included at 2× concentration. The gels were allowed to polymerize over 30 min followed by addition of 0.6 mL 10% FBS-RPMI. The gels were then allowed to contract over 24 h. At the end of the assay the plates were scanned, and the area of each gel was measured using ImageJ software with the circular lasso tool. Each experiment had a replicate of 5–8 and was repeated two times.

### 4.8. Statistical Analysis

Analyses were performed using Prism 7 for Mac (San Diego, CA, USA). Normality of data was assessed by the D’Agostino & Pearson normality test. Data comparisons were performed with one-way ANOVA for parametric data or Kruskal-Wallis test for non-parametric data. All data are presented as mean ± SEM. Values of *p* < 0.05 were deemed as significant.

## 5. Conclusions

In conclusion, our study evaluated the effects of CPDD and Pem, the first line chemotherapeutics, on different MPM cell lines representing the three different mesothelioma histological subtypes in 3D culture assays. The advantage of a 3D cell culture platform is that it can be used to assess the efficacy of adjuvant drugs in the context of MPM treatment.
